# Rats bred for low and high running capacity display alterations in peripheral tissues and nerves relevant to neuropathy and pain

**DOI:** 10.1002/brb3.780

**Published:** 2017-09-06

**Authors:** Michael A. Cooper, Megan M. Jack, Janelle M. Ryals, Page Hayley, Taylor Escher, Lauren G. Koch, Steven L. Britton, Shelby M. Raupp, Michelle K. Winter, Kenneth E. McCarson, Paige C. Geiger, John P. Thyfault, Douglas E. Wright

**Affiliations:** ^1^ Department of Anatomy and Cell Biology University of Kansas Medical Center Kansas City KS; ^2^ Department of Neurosurgery University of Kansas Medical Center Kansas City KS; ^3^ Department of Anesthesiology University of Michigan Ann Arbor MI; ^4^ Department of Molecular and Integrative Physiology University of Michigan Ann Arbor MI; ^5^ Kansas Intellectual and Developmental Disabilities Research University of Kansas Medical Center Kansas City KS; ^6^ Department of Pharmacology Toxicology and Therapeutics University of Kansas Medical Center Kansas City KS; ^7^ Department of Molecular and Integrative Physiology University of Kansas Medical Center Kansas City KS; ^8^ Research Service Kansas City Medical Center Kansas City MO

**Keywords:** aerobic fitness, DRG, inflammation, pain

## Abstract

**Introduction:**

Diet and activity are recognized as modulators of nervous system disease, including pain. Studies of exercise consistently reveal a benefit on pain. This study focused on female rats to understand differences related to metabolic status and peripheral nerve function in females.

**Methods:**

Here, we investigated parameters of peripheral nerve function relevant to pain in rats selectively bred for high (high‐capacity runners; HCR) or low endurance exercise capacity (low‐capacity runners; LCR) resulting in divergent intrinsic aerobic capacities and susceptibility for metabolic conditions.

**Results:**

LCR female rats have reduced mechanical sensitivity, higher intraepidermal nerve fiber density and TrkA‐positive epidermal axons, increased numbers of Langerhans and mast cells in cutaneous tissues, and a higher fat content despite similar overall body weights compared to female HCR rats. Sensory and motor nerve conduction velocities, thermal sensitivity, and mRNA expression of selected genes relevant to peripheral sensation were not different.

**Conclusions:**

These results suggest that aerobic capacity and metabolic status influence sensory sensitivity and aspects of inflammation in peripheral tissues that could lead to poor responses to tissue damage and painful stimuli. The LCR and HCR rats should prove useful as models to assess how the metabolic status impacts pain.

## 1 INTRODUCTION

Physical activity and exercise affect a number of metabolic parameters, however, not all individuals experience the same benefits from exercise. A multitude of genetic factors influence actions of physical activity and exercise (Bouchard et al., 1986), and these benefits vary among different tissues and organs. Low‐capacity running (LCR) and high‐capacity (HCR) rats are an experimental rat model designed to examine the underlying mechanisms by which high and low aerobic capacity impact susceptibility for a variety of chronic disease conditions and lifespan. LCR and HCR rats are commonly used in physiological studies designed to understand mechanisms and genetics associated with exercise, activity and disease risk (Geisser et al., 2008; Muncey et al., 2010). LCR and HCR rats are outbreed line generated from a founder population of male and female N:NIH stock rats based on intrinsic aerobic capacity assessed once at each generation by forced speed‐ramped treadmill running until exhaustion, after which animals are kept in sedentary housing (Koch & Britton, 2005). The metabolic differences between LCR and HCR rats have shed light on various aspects of activity and have proven useful in understanding how metabolic status affects disease. The LCR/HCR rat model has identified a genetic basis for the benefits of aerobic capacity and exercise on various organ systems including the heart, liver, skeletal muscle, lung, and brain (Hussain, Barbato, Koch, Metting, & Britton, 2001; Kirkton et al., 2009; Kivela et al., 2010; Koch & Britton, 2005; Murray et al., 2010; Wisloff et al., 2005).

There is growing interest about metabolic dysfunction and/or exercise impact the peripheral and central nervous system, particularly as it relates to neural disease (Cameron & Cotter, 1997; Chowdhury, Smith, & Fernyhough, 2013; Cooper, Kluding, & Wright, 2016; Farooqui, 2012). It remains unclear how the genetic differences underlying intrinsic aerobic capacity between LCR and HCR rats alter the peripheral nervous system. Previous studies have reported that LCR rats have increased fat mass and proinflammatory signaling in adipose tissue, as well as alterations in cholinergic anti‐inflammatory signaling (Bowden‐Davies et al., 2015; Su et al., 2012). Both are known to be important in peripheral nerve function and pain.

Here, we compared a number of pain‐ and neuropathy‐relevant features of the peripheral nervous system in female LCR and HCR rats. The selective breeding of the LCR and HCR strains results in differences in peripheral nerve sensitivity, cutaneous innervation, and composition of the dermis and epidermis. The nature of the differences in female LCR rats is consistent with known alterations in the periphery associated with poor outcomes in pain and peripheral nerve disease in humans. These findings in LCR rats suggest they could serve as novel model to explore the genetic features important in pain and abnormal sensory function associated with low aerobic capacity, which is a known risk for developing obesity and type 2 diabetes.

## 2 MATERIALS AND METHODS

### 2.1 LCR and HCR rats

The development of high‐ /low‐capacity rats (HCR/LCR) model displaying high and low intrinsic aerobic capacity has been previously described (Koch & Britton, 2001; Noland et al., 2007; Wisloff et al., 2005). Female rats 40–50 weeks of age were dual housed on a 12‐hr light cycle on a control chow diet [8604 (14% kcal fat, 54% CHO, 32% protein, 3.9 kcal/g), ENVIGO, Madison WI] throughout the period of analysis. All animal use was in accordance with NIH guidelines and conformed to protocols approved by the University of Kansas Medical Center Institutional Animal Care and Use Committee. All rats were examined at the University of Michigan prior to their shipment to the University of Kansas Medical Center for running distance, speed, time to exhaustion, and energy expenditure as previously described (Koch & Britton, 2001).

### 2.2 Body composition

Body composition to assess fat mass was measured by MRI using the EchoMRI‐100 (EchoMRI, Houston, TX). Fat mass and lean mass were analytically determined immediately prior to exsanguination. Animal weights were measured at the time of sacrifice allowing for both fat and lean mass to be reported relative to percent body weight.

### 2.3 Blood chemistry

At the time of sacrifice, blood was drawn from the chest cavity by cardiac puncture and allowed to clot for 25 min at room temperature then placed on ice. All samples were then spun at 3,000*g* for 10 min at 4°C and serum separated and frozen at −80°C until subsequent analysis was performed. Blood serum analysis (total protein, albumin, globulin, sodium, potassium, chloride, CO_2_, calcium, glucose, alkaline phosphatase, alanine aminotransferase, bilirubin, phosphorous, blood urea nitrogen, creatinine, cholesterol, triglycerides, and insulin) performed by Comparative Clinical Pathology Services LLC (Columbia, MO). All rats were removed from their food the previous night (12 hr prior to collection).

### 2.4 Behavioral testing

Animals were acclimatized and tested for mechanical and thermal sensitivity as previously described (Winter & McCarson, 2005). Thermal sensitivity latencies from the six applications were used to calculate the mean latency per animal and mean latencies were combined to calculate group means. Mechanical sensitivity was measured twice (1 week apart) 2 weeks prior to sacrifice, and the two testing days were averaged together to give one total mechanical sensitivity value.

### 2.5 Nerve conduction velocity measurements

Rats were anesthetized with an IP injection of 50 mg/ml phenobarbital sodium salt (Sigma, St. Louis, MO) and motor and sensory nerve conduction velocities were recorded. The left sciatic‐tibial motor conduction system was stimulated proximally at the sciatic notch and distally at the ankle via bipolar electrodes with supramaximal stimuli (9.9 mA) for 0.2 ms with low‐ and high‐pass filters of 20 Hz and 10 Hz, respectively. The latencies of the compound muscle action potentials were recorded via bipolar electrodes from the first interosseous muscle of the hind‐paw and were measured from the stimulus artifact to the onset of the negative M‐wave deflection. Motor nerve conduction velocity (MNCV) was calculated by subtracting the distal latency from the proximal latency and the result was divided into the distance between the stimulating and recording electrode. Hind limb sensory nerve conduction velocity (SNCV) was recorded in the digital nerve to the second toe by stimulating with a square‐wave pulse of 2.4 mA for a duration of 1.0 ms utilizing a low‐ and high‐pass filter of 3 Hz and 10 Hz, respectively. The sensory nerve action potential was recorded behind the medial malleolus. Ten responses were averaged to obtain the position of the negative peak. The maximal SNCV was calculated from the latency to the onset of the initial negative deflection and the distance between stimulating and recording electrodes.

### 2.6 Intraepidermal nerve fiber and langerhans cell measurements

Rats were exsanguinated following nerve conduction velocity measurements. Cutaneous tissue from the pad of the hind paw were collected, processed, and stained for IENF density (IENFD) as previously described (Jack, Ryals, & Wright, 2012). IENFD was expressed as number of fibers per millimeter of epidermis from a total of nine images per rat. The combined mean IENFD from each rat was used to calculate group means.

Langerhans cell measurement was performed using Langerin (E‐17) (1:1000; Santa Cruz Biotechnology Inc., Santa Cruz, CA) to visualize Langerhans cells. Fluorescent images were collected using a Nikon Eclipse 90*i* microscope using a 10× objective. NIH image J software was used to measure each dermal region. Langerhans cell density was expressed as number of Langerhans cells per millimeter of epidermis from a total of nine images per rat.

### 2.7 Mast cell quantification

Footpads were collected and processed as previously described for mast cell quantification (Fuentes, Pierce, O’Neil, & Christianson, 2015). After imaging a standardized region of interest was placed over all images using NIH image J software and all mast cells within the region of interest were counted manually.

### 2.8 Expression of mRNA encoding pain genes

RNA was extracted and cDNA synthesized as previously described (Jack, Ryals, & Wright, 2011). Primer sequences for ASIC3, CGRP, COMT, SCN9A, SCN10A, TrkA, TRPA1, TRPV1, and TRPV4 were created for rat sequences by Integrated DNA Technologies (Coralvile, IA). All reactions were performed in triplicate, and all mRNA levels were normalized to GAPDH. ΔΔCT values were used to calculate fold change and relative expression levels.

### 2.9 Statistical analyses

Results are presented as means ± SEM. Data were analyzed using unpaired t‐test and two‐factor ANOVA with post hoc comparisons using Fisher's test of least square difference where appropriate. Statistical significance was set at *p* < .05 and analyses were performed using GraphPad Prism 7 (GraphPad Software Inc., La Jolla, CA).

## 3 RESULTS

### 3.1 LCR rats have reduced aerobic capacity

**Figure 1:**
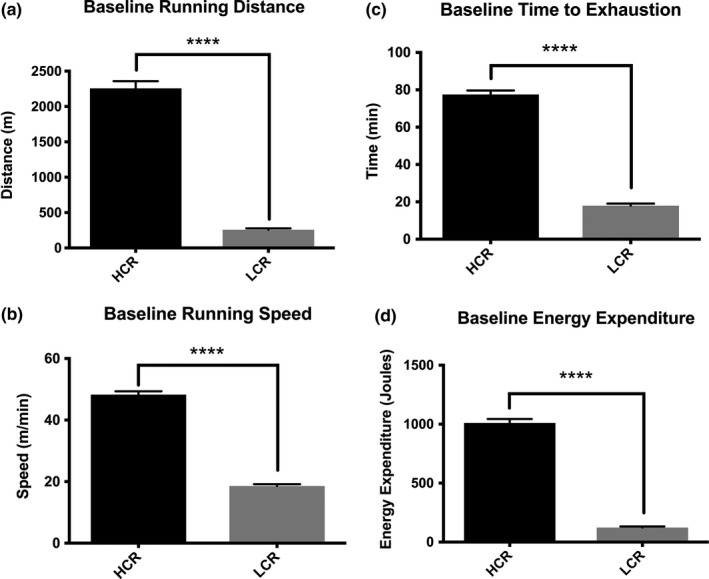
Animals classified as high‐capacity runners display increased abilities in all aspects of aerobic testing. Baseline testing of HCR and LCR animals shows increased distance, speed, running time, and energy expenditure in high capacity classified animals as compared to low‐capacity runners. (*n* = 18 for both groups) All data are presented as mean ± SEM. ****p<0.001

LCR and HCR rats were examined for running distance, speed, time to exhaustion, and energy expenditure on a single occasion at the University of Michigan prior to their shipment to the University of Kansas Medical Center. Consistent with this model, LCR rats displayed significantly reduced running distance (*p* < .0001), running speed (*p* < .0001), time to exhaustion (*p* < .0001), and energy expenditure (*p* < .0001) compared to HCR rats (Figure 1a–d).

### 3.2 LCR rats have more fat despite weighing the same as HCR rats

Female LCR and HCR rats had equal body weights at the time of sacrifice (LCR, 248.3 ± 4.559; HCR, 246.6 ± 5.725), though the percent of body weight due to fat versus lean mass differed between groups (LCR, 8.44% ± 0.6589; HCR, 5.33% ± 0.4328). LCR rats had significantly more of their mass distributed as fat (*p* = .0004) and a lesser amount due to lean mass (*p* = .003).

### 3.3 LCR rats have lower mechanical thresholds

**Figure 2:**
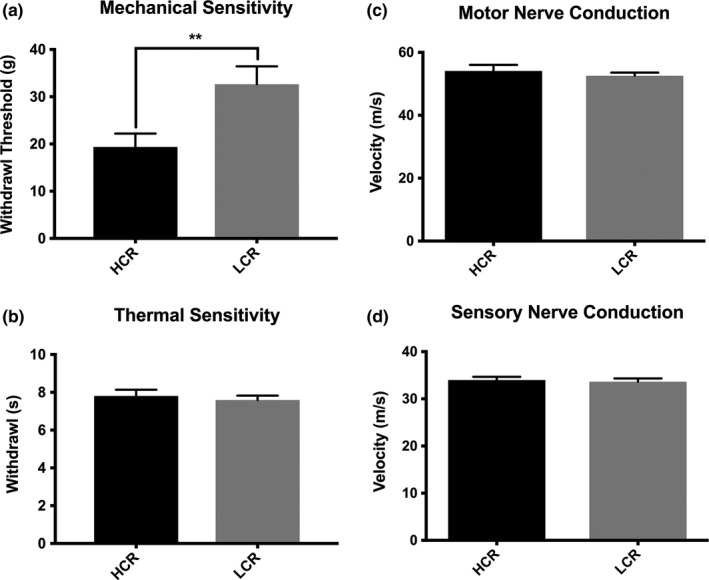
Mechanical sensitivity is reduced is LCR Females. (a) Mechanical sensitivity test displayed strain specific differences in basal sensitivity of LCR animals. (b) Thermal sensitivity testing showed no alterations in withdrawal latency between HCR and LCR groups. (c) Basal motor nerve conduction velocities show no strain differences between HCR and LCR animals. (d) Basal sensory nerve conduction velocities are unaltered between HCR and LCR animas. All data are presented as mean ± SEM;* n* = 18 for all groups. **p<0.01

Mechanical withdrawal thresholds of the hind paws were significantly reduced in LCR rats, as compared to their HCR counterparts (*p* = .008, Figure 2a). There were no differences in thermal latencies between LCR and HCR rats (Figure 2b). Conduction velocities for both motor and sensory neurons for both LCR and HCR appeared comparable with no differences were noted between LCR and HCR rats related to conduction velocities (Figure 2c and d).

### 3.4 LCR rats have poor indicators of blood chemistry

Analyses of a range of blood biomarkers are displayed in Table 1. LCR rats display increased potassium (*p* = .005), calcium (*p* = .027), alkaline phosphatase (*p* < .0001), and triglycerides (*p* = .030). All remaining blood serum markers (total protein, albumin, globulin, sodium, chloride, total CO_2_, glucose, alkaline aminotransferase, gamma‐glutamyltransferase, total bilirubin, phosphorus, blood urea nitrogen, creatine, cholesterol, and insulin) were not significantly different between LCR and HCR rats.

**Table 1:** LCR rats display few variances from HCR rats in serum markers

	HCR‐fasted	LCR‐fasted
Total protein (g/dl)	6.05	6.11
Albumin (g/dl)	3.40	3.43
Globulin (g/dl)	2.65	2.68
Sodium (mEq/L)	138.30	140.20
Potassium (mEq/L)	3.23	3.78^b^
Chloride (mEq/L)	98.74	99.52
Total CO2 (mEq/L)	25.70	27.80
Calcium (g/dl)	9.37	10.24^a^
Glucose (mg/dl)	180.00	169.50
Alkaline Phosphatase (U/L)	51.20	101.6^c^
Alanine aminotransferase (U/L)	58.20	75.60
Gamma‐glutamyltransferase (U/L)	<3	<3
Total bilirubin (mg/dl)	0.11	0.10
Phosphorus (mg/dl)	5.01	4.69
Blood urea nitrogen (mg/dl)	18.40	19.50
Creatinine (mg/dl)	0.19	0.26
Cholesterol (mg/dl)	77.40	83.70
Triglycerides (mg/dl)	50.20	78.5^a^
Insulin (ng/ml)	1.05	2.17

Serum blood analysis from 12‐hour fasted HCR and LCR animals. LCR animals have statistically increased potassium, alkaline phosphatase, calcium, and triglycerides, while no other markers were statistically different between LCR and HCR animals.

a *p* < .05.

b *p* < .01.

c *p* < .0001.

### 3.5 LCR rats have a higher density of epidermal axons

**Figure 3:**
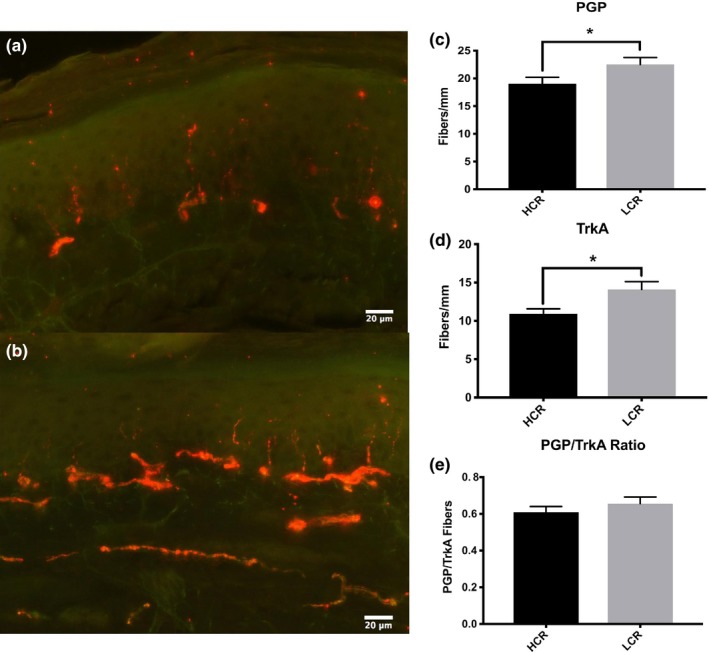
LCR animals display increased cutaneous innervation. Merged representative images of double immunofluorescent staining for the pan‐neuronal markers PGP9.5 and TrkA. (a) HCR (b) LCR (c) LCR animals show increased nerve fiber density in the hind paw skin. (d) LCR animals display increased peptidergic nerve fiber density in the hind paw skin. (e) The ratio of TrkA/PGP9.5 shows no difference between groups when accounting for total innervation. All data are presented as mean ± SEM;* n* = 6 for all groups. *p<0.05

Total C‐fiber axonal density was increased in the epidermal footpad of LCR rats (LCR = 23 fibers/mm^2^; HCR = 19 fibers/mm^2^, *p* = .047, Figure 3c). The axonal subset of C‐fibers, which express the tyrosine kinase receptor TrkA, and respond to NGF were significantly increased in LCR rats (LCR = 14 fibers/mm^2^; HCR = 11 fibers/mm^2^, *p* = .110, Figure 3d). The ratio of TrkA fibers to total PGP‐9.5 + fibers was similar between the two groups (Figure 3e).

### 3.6 LCR rats have increased langerhans cells and mast cells in cutaneous tissues

**Figure 4:**
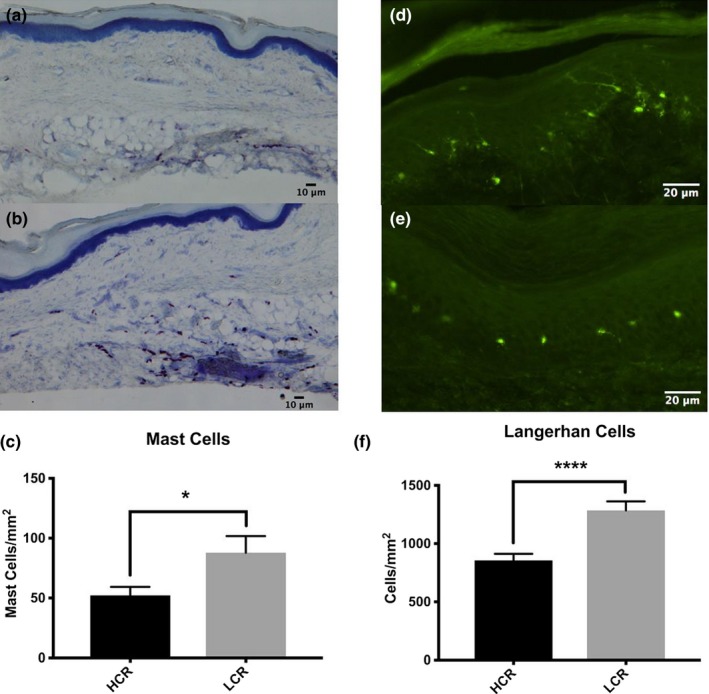
LCR animals display increased mast and Langerhan cell density. Representative images of toluidine blue staining for mast cells. (a) HCR (b) LCR (c) LCR animals show increased mast cell density in the hind paw skin. All data are presented as mean ± SEM;* n* = 5 for all groups. Representative images of immunofluorescent staining for Langerin (E‐17). (d) HCR (e) LCR (f) LCR animals show increased Langerhan cell density in the hind paw skin. All data are presented as mean ± SEM;* n* = 6 for all groups. *p<0.05. ****p<0.001

Quantification of the number of Langerhans cells in the epidermis revealed that LCR rats have significantly higher number of Langerhans cells compared to HCR animals (*p* < .0001, Figure 4c). In addition, the density of mast cells was increased in the dermis of LCR rat hind paws relative to HCR rats (*p* = .024, Figure 4f).

### 3.7 LCR and HCR have similar expression of pain genes

Analysis of gene expression in the lumbar DRG of LCR and HCR rats revealed no significant strain‐related differences in the levels of any of the mRNAs examined (ASIC3, CGRP, COMT, SCN9A, SCN10A, TrkA, TRPA1, TRPV1, and TRPV4).

## 4 DISCUSSION

Metabolic syndrome is a medical epidemic in Western society that is characterized by a constellation of risk factors including insulin resistance, elevated triglycerides, hypertension, and central obesity. The metabolic syndrome is known to increase risk for type 2 diabetes and cardiovascular disease (Welty, Alfaddagh, & Elajami, 2016). Numerous studies have linked both prediabetes and metabolic syndrome in animal models and human patients with the development of sensor motor polyneuropathy (Callaghan et al., 2016). Preclinical modeling of polyneuropathy has been challenging and improved animal models encompassing the intricacies of human disease are vitally needed (Biessels et al., 2014; Mogil, 2009).

LCR and HCR rats reflect the complex nature of metabolic syndrome through extensive breeding for traits known to characterize both a healthy and poor metabolic state. The two strains can be divided based on their low and high health risk factors determined by their propensity to run greater or shorter distances based on their divergence in intrinsic aerobic capacity (Koch & Britton, 2005). To date, only male LCR and HCR rats have been studied related to peripheral nerve function (Geisser et al., 2008; Muncey et al., 2010), despite a higher incidence of chronic pain conditions in female patients (Dodds et al., 2016; Melchior, Poisbeau, Gaumond, & Marchand, 2016).

HCR rats displayed increased aerobic function and decreased fat mass consistent with prior characterization of the strain, whereas LCR rats displayed decreased aerobic function and increased fat mass (Koch & Britton, 2005; Ren et al., 2013). Female LCR and HCR rats were not different in their overall weights. Prior studies have shown clear differences in body weight in female rats, though less pronounced compared to their male counterparts (DeMarco et al., 2012; Ritchie et al., 2013). It is likely that examination of aged female rats would have revealed differences in overall body weight. In this study, body weight does not appear to be a confounding factor in our observations; differences in fat mass distribution is a more plausible modulator of peripheral nerve function rather than overall weight.

This study is the first to examine the serum of female HCR and LCR animals across a number of metabolic markers. While potassium levels were statistically different, the differences were not physiologically relevant since values less than 6 mEq/L are considered normal (Charles, 1982). Similarly, calcium levels fall between 9 and 11 mg/dl, and the values observed are not likely physiologically different between LCR and HCR animals (Charles, 1982). Circulating triglycerides, although significantly higher in LCR rats, were still within reported ranges (Charles, 1982). Alkaline phosphatase was also elevated in LCR animals, however, levels remained below the maximum of reported ranges (maximum of 131 U/L) (Charles, 1982). The serum analysis did not identify any differences that explain the anatomical and behavioral differences between LCR and HCR rats.

### 4.1 Cutaneous mechanical sensitivity

Female LCR and HCR rats do not demonstrate strain‐related baseline differences in thermal sensitivity. Previous studies with male LCR and HCR rats demonstrated differences in thermal sensitivity between the strains with LCR mice having lower paw withdrawal latencies (Geisser et al., 2008). Conduction velocities of motor and sensory nerves are an important clinical indicator of diabetic neuropathy and myopathy (Dorfman & Bosley, 1979; Kimura, 2013). Here, no differences were observed between either sensory or motor nerve conduction velocity between LCR and HCR rats.

Male LCR and HCR rats do not demonstrate baseline mechanical sensitivity differences (Muncey et al., 2010). Female LCR and HCR rats demonstrated different baseline responses to mechanical withdrawal testing. This suggests, LCR rats have some level of insensitivity to mechanical stimuli in comparison to HCR rats. The perception of sensory input is likely altered in female LCR rats since the peripheral nervous system remains structurally intact with normal nerve conduction velocities. This baseline structural similarity between the two strains is important to establish, as the hallmarks of peripheral neuropathy are reduced conduction speeds, loss of myelination, and altered sensory sensitivity. This lack of structural differences leads us to hypothesize the differences in mechanical sensitivity are related to a chronic inflammatory state as a result of the metabolic syndrome occurring in LCR rats.

Models of type 2 diabetes presenting with common signs of metabolic syndrome such as increased body weight, fat mass, and blood glucose, often also show systemic inflammation and heightened sensitivity (Drel et al., 2006). Previous work examining the role of inflammation in altered sensitivity shows that with profoundly increased inflammation there can be an alteration in both thermal and mechanical sensation (Andrew & Greenspan, 1999; Kidd & Urban, 2001). Examination of painful areas has revealed that mast cells are a key marker of inflammatory changes in pain states and offer a key correlation between their density and resulting sensitivity (Heron & Dubayle, 2013). On the basis of this information, we suggest the chronic inflammatory environment present in the LCR rat is a state that is primed to drive lower sensory thresholds and allodynia with alterations in diet, exercise, or disease. The LCR rat, given its baseline proinflammatory physiologic state, likely has compensatory central and peripheral modulators of allodynia that result in reduced sensitivity and higher thresholds. We hypothesize alterations to their physiologic state through diet, injury, or disease state will overwhelm their compensatory strategies to mitigate allodynia and promote sensory abnormalities. Hence, this rat model lends itself nicely to the study of polygenic features seen in patients with neuropathy but have been challenging for inbred or genetic animal models to accurately model.

Genetic differences underlying mechanical and thermal sensitivity have been well established by our laboratory and others (Jack et al., 2012; Mogil, 2012). Some have also implicated sex differences with the variability in sensory thresholds; despite this, majority of studies generally utilize male mice for studies (Craft, Mogil, & Aloisi, 2004; Klein et al., 2015). Genetic approaches using inbred mouse strains have identified a number of mRNAs that are altered in their expression associated with pain genetics (Lariviere & Mogil, 2010; Mogil, 2012). On the basis of this information, we investigated a select number of known genes involved with nociceptive transmission in the LCR‐HCR models. No strain differences were seen in the mRNAs studied that are known to have a role in nociception, which suggests at least in the expression of the genes studied, there is no underlying physiologic difference in nociceptor properties.

### 4.2 Intraepidermal nerve fiber density

IENFD is a valuable tool to visualize sensory innervation of the epidermis by small unmyelinated C‐fibers (England et al., 2009; Smith et al., 2005). These C‐fibers are responsible for transmitting mechanical, thermal, and noxious stimuli from the epidermis and are impacted by the metabolic status in animals and humans (Woolf & Salter, 2000). Reductions in IENFD correlate with small fiber damage and sensory neuropathies (Lauria et al., 1999; Pittenger et al., 2004). Previous work by our group and others has displayed that IENFD is altered by changes in diet and exercise (Callaghan et al., 2016; Groover et al., 2013; Smith et al., 2006). Here, female LCR rats demonstrated a significant increase in the density of PGP‐9.5 + intraepidermal nerve fibers compared to HCR rats. It should be reminded that alterations between groups are not due to physical activity effects on the skin, as these animals are not exercised after initial baseline examination. The LCR rats also had increased number of fibers expressing TrkA, the high affinity receptor for NGF. TrkA‐positive axons play an important role in pain transmission (Costigan & Woolf, 2000; Mantyh, Koltzenburg, Mendell, Tive, & Shelton, 2011). However, the overall percentage of TrkA fibers was similar between groups. Studies have established allodynia and reduced mechanical thresholds are associated with increased TrkA fibers (Cheng, Dauch, Hayes, Hong, & Feldman, 2009; Cheng, Dauch, Hayes, Yanik, & Feldman, 2012; Groover et al., 2013). This increase in IENF and increase in TrkA+ fiber density may predict that LCR rats display a greater response to evoked allodynia and are at risk for longer duration of heightened sensitivity.

### 4.3 Langerhans cells

Langerhans cells are transient dendritic antigen‐presenting cells located in the epidermis. They play a critical role in the epidermal immune response and are also found within peripheral nerve bundles that innervate the plantar and palmar surfaces (Dauch et al., 2013). Langerhans cells function using mechanisms similar to macrophages and respond to similar signaling agents such as inflammatory cytokines and calcitonin gene‐related peptide (Hosoi et al., 1993; Stingl, Katz, Clement, Green, & Shevach, 1978). These cells are found in close proximity to regulatory T cells and effector memory T cells, again highlighting a strong antigen‐response role in the epidermis (Seneschal, Clark, Gehad, Baecher‐Allan, & Kupper, 2012). Previous studies have demonstrated that Langerhans cells density correlates strongly with intraepidermal nerve fiber density and changes in mechanical sensitivity associated with allodynia (Doss & Smith, 2012, 2014). In mice depleted of Langerhans cells using diphtheria toxin, mechanical sensitivity increased as a result of the loss of Langerhans cells (Doss & Smith, 2014). Similar to this result, female HCR rats in this study display decreased epidermal Langerhans cells as compared to their LCR counterparts and decreased mechanical sensitivity thresholds.

### 4.4 Mast cells

Similar to Langerhans cells, mast cells play a significant role in innate and adaptive immunity; however, they modulate neuropeptides involved in sensory processing and signaling (Aich, Afrin, & Gupta, 2015; Madva & Granstein, 2013). Mast cells have a well‐established neuroimmune response through the reaction to inflammatory signaling and subsequent synthesis and release of neurotrophins highlighting an important molecular marker of peripheral nerve inflammatory response (Bienenstock et al., 1987; Leon et al., 1994). Together these neurophysiological and molecular markers indicate peripheral nerve function and health status. Mast cell degranulation releases a number of molecules important in nociceptive signaling and degranulation is known to be an important contributor to pain (Fuentes et al., 2015; Pierce et al., 2016). Female LCR rats have significantly higher numbers of mast cells in epidermis and dermis of the hind paw compared to female HCR. Again, this may be reflective of their inflammatory state, which could contribute to the development of sensation abnormalities.

In conclusion, female LCR and HCR rats demonstrated differences in mechanical, but not thermal sensitivity. The strains did not have altered gene expression for common markers, ion channels, and receptors of nociceptors. Female HCR and LCR rats had similar nerve conduction velocities and proportion of intraepidermal nerve fiber types in the hind paw. Female LCR rats show increased inflammatory cells within the peripheral nervous system that could increase allodynia in diseased states. In order to understand sex differences in allodynia and analgesia, female HCR and LCR were used to determine baseline characteristics of the peripheral nervous system in preparation for future studies investigating disease models affecting cutaneous sensation.
